# Composition and distribution of bacterial communities and potential radiation-resistant bacteria at different elevations in the eastern Pamirs

**DOI:** 10.3389/fmicb.2024.1427806

**Published:** 2024-06-19

**Authors:** Jing Zhu, Hui-Nan Wang, Qi-Yong Tang, Mei-Ying Gu, Zhi-Dong Zhang

**Affiliations:** Xinjiang Laboratory of Special Environmental Microbiology, Institute of Applied Microbiology, Xinjiang Academy of Agricultural Sciences, Urumqi, China

**Keywords:** bacterial communities, elevation gradient, radiation resistance, environmental adaptation, Pamir plateau

## Abstract

Altitude and ultraviolet (UV) radiation may affect the community composition and distribution of microorganisms in soil ecosystems. In this study, 49 soil samples from 10 locations were collected from different elevations on the eastern Pamir Plateau and analyzed for soil microbial community structure and function using high-throughput sequencing. The results showed that soil samples from different elevations of the eastern Pamir Plateau contained 6834 OTUs in 26 phyla and 399 genera. The dominant phyla common to different elevations were *Actinobacteria*, *Proteobacteria*, *Bacteroidota*, *Acidobacteriota*, and *Gemmatimonadota*. The dominant genera were *Rubrobacter*, *Sphingomonas*, *Nocardioides*, and *Solirubrobacter*. Species richness increased slightly with elevation, and there were significant differences in community composition between the elevations. Elevation and UV exposure are important factors that drive changes in bacterial communities. The results of the KEGG pathway showed that drug resistance, antineoplastic, aging, replication, and repair were enhanced and then slightly decreased with increasing elevation. Bacterial communities at different elevations were rich in radiation-resistant microorganisms, and the main genera were *Rubrobacter*, *Sphingomonas*, *Nocardioides*, *Pontibacter*, and *Streptomyces*. The findings have shown the composition and distribution of bacterial communities at different elevations on the Eastern Pamir Plateau. Potentially radiation tolerant microbial species were also examined. The results are of considerable importance for the succession of bacterial microorganisms in the plateau region, the study of radiation tolerant bacterial germplasm resources, and the application of biofunctionality.

## 1 Introduction

Plateau ecosystems are among the most important terrestrial ecosystems. Their climate, vegetation, and soil properties vary widely over short spatial distances and altitudinal gradients (McCain, 2010). Francos et al. (2021) found that elevation and total nitrogen (TN) are two important factors affecting organic matter (OM) in surface and subsurface soil layers. For areas above 3000–4000 m elevation, fractional vegetation cover has the strongest response to precipitation and temperature (Zhang et al., 2019). Owing to low temperatures and soil aridity or salinization in plateau areas, such habitats can reduce soil productivity or disrupt soil system function. A specific pattern of soil microbial diversity and taxonomic composition is formed. In this context, environmental stress plays a key role in soil microbial community changes (Khomutovska et al., 2021; Ji et al., 2022). Elevation has multiple response mechanisms in plateau ecosystems. These relationships may reflect the different feedback mechanisms and processes of ecosystems in response to environmental gradients (Bagousse-Pinguet et al., 2019).

The Pamir Plateau is one of the world’s top ten plateaus with massive mountains and precipitous terrain. It is the center of the concentrated development of alpine glaciers and is an intense natural environment with extremely arid and cold climates. This climatic region harbors a wide range of microbial resources (Yang and Yan, 2008; Yang et al., 2018; Barandun et al., 2021; Zhang et al., 2021). To date, domestic and international research on microorganisms in the Pamir region has focused on the glaciers, rocky substrates, and mountainous deserts. Khomutovska et al. (2017, 2020) studied endolithic microorganisms from the cold desert ecosystems of Eastern Pamir. Their community composition and structure were influenced by the microhabitat structure and associated microenvironmental conditions. Li et al. (2015) showed that plateau glacier habitats are nutrient-poor and a wide range of their microorganisms are better suited to oligotrophic survival environments. However, to date, there have been relatively few studies conducted on the composition of bacterial communities and potential radiation-tolerant bacteria in soil samples collected from different elevations in the East Pamir region.

The Pamir Plateau is exposed to high solar radiation. The highest solar radiation intensity in the region is 943.2 kW h/m^2^ in summer with the lowest value being 301.6 kW h/m^2^ in winter (Safaraliev et al., 2020). This is an extreme habitat that may contain abundant extreme microbial resources that can be used. Microbes that can survive high radiation doses are known as radiation-tolerant microorganisms. They are mainly found in desert soils, plateau lakes, radiation-polluted areas, and Antarctica (Fredrickson et al., 2004; Paulino-Lima et al., 2016; Portero et al., 2019; Monsalves et al., 2020). They have extreme resistance to DNA-damaging environments, that is, ionizing radiation and UV rays, mutagens, and desiccation environments (Narasimha and Basu, 2021). They possess highly efficient and precise DNA repair systems (Agapov and Kulbachinskiy, 2015) and antioxidant systems (Qi et al., 2020). Developing such microbial resources and studying their DNA damage repair mechanisms are of considerable importance for exploring the molecular mechanisms of DNA repair. They also play a substantial role in promoting the development of DNA technology. Meanwhile, the research and development of radiation-resistant microorganisms have a range of considerable potential applications in fields such as environmental engineering (Shang et al., 2021), medicine (Singh and Gabani, 2011; Gabani and Singh, 2013), and agriculture (Pan et al., 2009).

To date, relatively few studies have been conducted on the composition of bacterial communities at different elevations on the Pamir Plateau. Although there has been an increase in research on radiation-tolerant microorganisms, the use of radiation-tolerant microbial resources remains limited. The specific habitat of the Pamir Plateau is likely to have abundant radiation-tolerant microorganisms. In this study, we used high-throughput sequencing technology to determine the composition and distribution of bacteria at different elevations in East Pamir. We also examined the correlation between environmental factors and species preferences and investigated typical strains of radiation-tolerant bacteria; this would lay the foundation for the acquisition and use of radiation-tolerant resources.

## 2 Materials and methods

### 2.1 Sampling locations and methods

This study was conducted on the eastern Pamir Plateau (38°–41°N, 73°–76°E), China. Samples were collected at three elevations, with 49 soil samples from 10 sampling locations selected based on the geomorphology of the sampling sites (Table 1). Among them, the low elevation range was 1400–2400 m with an average elevation of 2231 m, including TH (Tokaji Aketedu County, shade), TS (Tokaji Aketedu County, sunny), and WQ (Wuqia County). The middle elevation range was 2400–3400 m with an average elevation of 3146 m, including G2 (Gaizi Bridge 2), G1 (Gaizi Bridge 1), B (Baisha Lake), and S7 (Subash Seventh Bridge). The high elevation range was 3400–4400 m with an average elevation of 3829 m including WH (Wakhan Corridor), MT (204 Mustag Iceberg Base), and SB (Subash Daban). Information on the elevation, temperature, moisture, coordinates, and UV radiation dose was collected at each location. After selecting a sample point, parallel samples were randomly selected at four points within a 50–100 m radius. To collect the soil samples, a surface layer of approximately 2 cm was removed, and soil samples were collected at a depth of 0–20 cm. Approximately 1 kg of soil was collected for each sample, placed in a self-sealing bag, and stored in the laboratory at 4°C. Parallel soil samples were mixed from each sampling site. Roots, stones, and other debris were removed, before 200 g of the samples were weighed for air-drying, They were then sent to the Testing Center of the Xinjiang Academy of Agricultural Sciences, Xinjiang Academy of Agricultural Sciences (Xinjiang, China) for physicochemical property analysis.

**TABLE 1:** Sampling information from the East Pamir region.

Sample	Site	Coordinates
Low altitude (1300–2400 m)	TH (Tokaji Aketedu County Shade)	75°29′34″E, 38°51′59″N
	TS (Tokaji Aketedu County Sunny)	75°24′55″E, 38°48′27″N
	WQ (Wuqia County)	73°59′5″E, 39°43′49″N
Medium altitude (2400–3400 m)	G2 (Gaizi Bridge 2)	75°11′55″E, 38°46′14″N
	G1 (Gaizi Bridge 1)	75°8′45″E, 38°44′54″N
	B (Baisha Lake)	75°1′6″E, 38°43′52″N
	S7 (Subash Seventh Bridge)	74°58′23″E, 38°58′23″N
High altitude (3400–4200 m)	WH (Wakhan Corridor)	75°20′30″E, 37°11′2″N
	MT (204 Mustag Iceberg Base)	74°58′1″E, 38°21′20″N
	SB (Subash Daban)	74°54′59″E, 38°16′8″N

A standard soil test series (NY/T 1121) was conducted. Organic matter (OM) was determined using the K_2_Cr_2_O_7_ oxidation method. The total nitrogen (TN) was measured using the Kjeldahl method. Available nitrogen (AN) was determined using the sulfate extraction method. Available phosphorus (AP) was detected using the hydrochloric acid–ammonium fluoride extraction–molybdenum antimony colorimetric method. Available potassium (AK) was detected using the ammonium acetate extraction–flame photometric method. Soluble salt (Salt) was detected using the mass method. The pH was determined using a potentiometric method.

### 2.2 DNA extraction and sequencing

Total soil DNA was extracted from a 0.5 g soil sample using the PowerSoil DNA Isolation Kit (Tiangen Biochemical, Beijing, China), according to the manufacturer’s instructions. DNA quality and quantity were assessed using a UVZ2550 spectrophotometer (Shimadzu Corporation, Kyoto, Japan) and 1% agarose gel electrophoresis, respectively. The samples were sent to Novogene Bioinformatics (Beijing) for amplification and sequencing of the V3–V4 regions of the 16S rRNA gene with standard Illumina 16S primers (338F:5′-ACTCCTACGGGGAGGCAGCA-3′,806R:5′-GGACTACHVGGGGTWTCTAAT-3′). Before amplification, tag sequences were added to the 5′ of downstream primers to distinguish between different samples.

### 2.3 Bioinformatics analysis

Quality filtering of downstream data from the Illumina MiSeq sequencing platforms was performed using QIIME software (version 1.9.1). Using UCLUST (version 1.1.579) software to cluster all the clean reads from all the samples, the sequences were clustered into operational taxonomic units (OTUs) by default with 97% identity. Species annotation analysis was performed using the Mothur method with the SSUrRNA database of SILVA132 with the threshold set at 0.8–1. Shannon, Simpson, Chao1, and other diversity indices were calculated using QIIME software. Redundancy analysis (RDA) was used to evaluate the correlation between the community structure and environmental variables using CANOCO (version 5.0, Microcomputer Power, Ithaca, N.Y., USA). Principal co-ordination analysis (PCoA) is a multivariate statistical technique used to explore and visualize the similarities or differences among samples and was performed using Wekemo BioIncloud (Gao et al., 2024). Co-occurrence network was constructed to predict interactions among members within bacterial communities. The frequencies of co-occurrence association (with | rho| > 0.7, *p* < 0.001) presented were recorded to reveal the association persistency among communities. Spearman’s *rho* statistic is used to estimate correlation with function *cor.test* in “stats” packagee (R Development Core Team, 2016). The co-occurrence networks were visualized with “igraph” package (Csardi and Nepusz, 2006). Network characteristics were determined using functions in “bipartite” package (Dormann et al., 2009). PUCRUSt2 predicts gene by link the ASV/OTU sequences to references genomes in PUCRUSt2 database. PUCRUSt2 database contains KEGG annotation informations of these reference genomes. PUCRUSt2 database meas built-in database of PICRUSt2 software (Douglas et al., 2020). We identified importance of species (variable) points by the randomforest R (Version 3.2.1). Cross-validation was performed by the “rfcv” function for selecting appropriate features. The “varImp-Plot” function was used to show the importance of features in the classification. Community composition maps were plotted using OriginPro 2021 software. IBM SPSS Statistics software for Windows (version 27.0, IBM Corp., Armonk, N.Y., USA) was used to analyze the differences between treatments. *p* value < 0.05 was considered significant.

## 3 Results

### 3.1 Bacterial community cluster analysis

**FIGURE 1:**
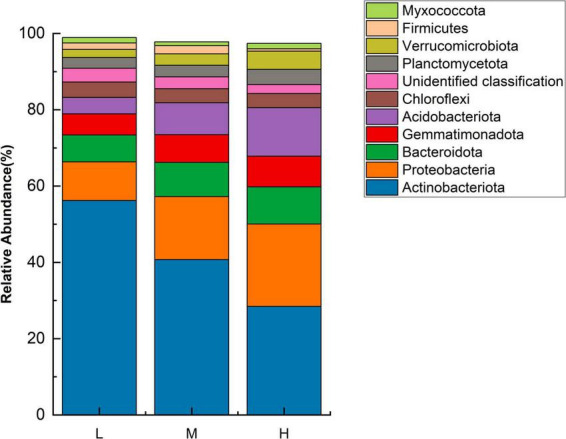
Relative abundance of major bacterial phyla at different elevations (*n* = 3) in the Eastern Pamir region.

Based on the sequencing results from 16S rRNA gene amplicons, the eastern Pamir Plateau soil samples from different elevations contained 6834 OTUs belonging to 26 phyla, 71 classes, 142 orders, 212 families, and 399 genera. Unidentified species were highly abundant. The bacterial communities at different elevations on the Pamir Plateau were significantly different at different taxonomic levels. Across all the samples, the explanation for a large proportion of the phyla was as follows. *Actinobacteriota* (56.21–28.45%) was the predominant phylum, followed by *Proteobacteria* (10.12–21.59%), *Bacteroidota* (7.04–9.73%), *Acidobacteriota* (4.30–12.72%), and *Gemmatimonadota* (5.55–8.06%). The top ten dominant phyla in terms of abundance were the same at both low and middle elevations. However, there were differences in relative abundance, with only *Myxococcota* being more abundant than *Firmicutes* at high altitudes, representing a difference between the low and middle elevations. In the three elevation ranges, the *Actinobacteriota* abundance decreased with increasing elevation. Meanwhile, the abundances of *Proteobacteria*, *Bacteroidetes*, *Gemmatimonadota*, and *Acidobacteria* increased with increasing elevation (Figure 1). This indicates that the four phyla with increased abundance are better suited for higher elevation habitats.

**FIGURE 2:**
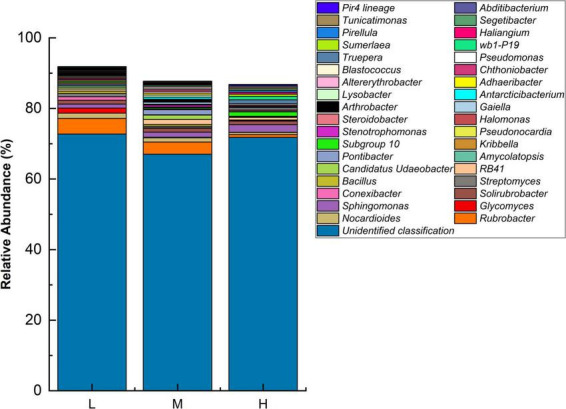
Relative abundance of major bacterial genera at different elevations (*n* = 3) in the Eastern Pamir region.

At the genus level, the highest abundances of unidentified genera were observed at all three elevations. The dominant genera identified were *Rubrobacter*, *Sphingomonas*, *Nocardioides*, and *Solirubrobacter*. There were differences in the dominant genera existed at different elevations. The low elevation dominant genera were *Rubrobacter* (4.43%), *Nocardioides* (1.47%), and *Glycomyces* (1.46%). The medium elevation dominant genera were *Rubrobacter* (3.46%), *Sphingomonas* (1.44%), and *Pontibacter* (1.44%). At the higher elevation, the dominant genera were *Sphingomonas* (2.15%), *Subgroup 10* (1.25%), and *Solirubrobacter* (1.02%). With increasing elevation, the relative abundances of *Rubrobacter* and *Nocardioides* decreased, *Sphingomonas* increased, and *Solirubrobacter* remained stable (Figure 2).

### 3.2 Species diversity analysis

The alpha diversity indices for bacterial communities in the soil samples from different elevations were analyzed. This included the Chao1 observed_features index for species richness, faith_pd for phylogenetic diversity, and the Shannon and Simpson indices for species diversity. The species richness and diversity indices were the lowest in the low elevation samples, significantly different from those in the middle and high elevations (Table 2). In contrast, diversity was highest at higher elevations but was not significantly different from species diversity at mid-elevations. Species diversity and richness indices increased with increasing elevation, indicating that higher elevation areas had higher species richness and diversity.

**TABLE 2:** Bacterial alpha diversity indices at different elevations on the Eastern Pamir Plateau.

Group	Chao1	faith_pd	Observed_features	Shannon	Simpson
L	1136.43 ± 156.45b	78.95 ± 7.50b	1130.47 ± 155.91b	8.79 ± 0.48b	0.9937 ± 0.0033b
M	1354.22 ± 165.45a	86.39 ± 7.07a	1347.111 ± 166.34a	9.22 ± 0.45a	0.9955 ± 0.026ab
H	1414.14 ± 138.44a	90.21 ± 7.43a	1407.87 ± 138.00a	9.49 ± 0.23a	0.9967 ± 0.0008a

The above data represent the mean ± standard deviation (n = 3). Different letters (a, b) indicate significant differences between the data (Duncan test, *p* < 0.05).

**FIGURE 3:**
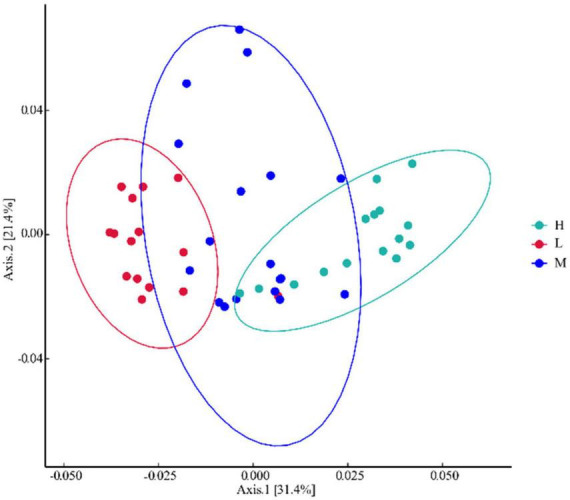
Principal coordination analysis of bacteria at different elevations in East Pamir.

Bacterial Alpha diversity and taxonomic composition analyses were performed using the community clustering approach of principal coordination analysis (PCoA). Unweighted UniFrac Bray–Curtis PCoA analysis showed that 31.4% of the total variation was explained by axis 1 and 21.4% by axis 2 (Figure 3). The low- and high-evaluation samples were significantly separated spatially, indicating a low similarity and high specificity of the community composition between the two samples. Middle elevation samples overlapped with all other samples, suggesting a similar species community composition between low and middle elevations, and between middle and high elevations.

### 3.3 Community-specific OTUs and core microbiome

The core microbiome has been defined in different ways (Sun et al., 2023). Turnbaugh et al. (2007, 2009) first proposed the core microbiome concept as “all taxa that are common to the microbiomes in all or the vast majority of habitats”. Delgado-Baquerizo et al. (2018) and Shade and Stopnisek (2019) defined core microbiome based on abundance-occupancy distributions that include highly abundant and ubiquitous taxa. In conclusion, core members is crucial to understanding the assembly mechanism of communities and identifying functionally important community members.

**FIGURE 4:**
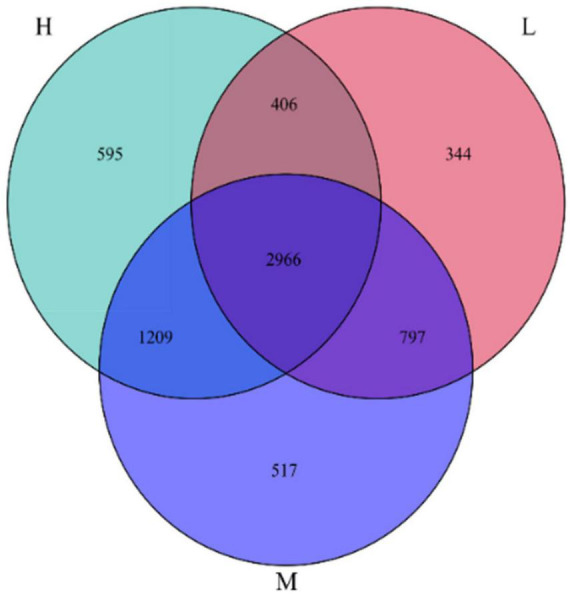
Venn diagram of the number of OTUs of samples at different elevation in the eastern Pamir Plateau

Based on a 97% similarity level, groups were set up at elevation intervals to obtain the OTUs data for each group of samples. The results of the Venn diagram for 6834 OTUs from 48 soil samples from the Pamir Plateau region are shown in Figure 4. There were 2966 identical OTUs at the three elevations, which accounted for 43.4% of the total OTUs. This indicated the presence of a core microbiome in the soil samples from different elevations. Among the total 2966 OTUs, the OTUs with higher relative abundance, that is, a relative abundance greater than 1%, were annotated as Sphingomonadaceae_B_OTU_17858, *Rubrobacter*, and Micrococcaceae_B_OTU_1232. These results suggest that these taxa have adapted well to the Pamir Plateau ecosystem and can survive in soil environments at different elevations.

There were substantial differences in the OTUs at different elevations. The numbers of OTUs specific to low, medium, and high elevations were 344, 517, and 595, respectively, accounting for 5.03, 7.57, and 8.71% of the total OTUs, respectively. This indicates that there were specific microbial communities in the soil samples at different elevations with a minor percentage. However, they were sensitive to elevation changes.

### 3.4 Environmental factors and soil physical and chemical properties analysis

The soil physicochemical properties were analyzed at three different elevations, and the results are shown in Table 3. The UV intensity increased with elevation, but the change was not significant between middle and high elevations. The soil temperature decreased from 25.7°C to 17.53°C with elevation. The OM increased from 4.63 g kg^–1^ to 12.03 g kg^–1^ with elevation. Soil AP content and pH increased with elevation. AN increased, and then decreased with altitude. The Salt initially increased and then decreased. TN content was significantly higher at high elevations than at low and middle elevations. Soil moisture and available potassium (AK) did not change significantly. Overall, the soil samples from different elevations in the eastern Pamir Plateau were nutrient-poor, arid, low-temperature alkaline soils.

**TABLE 3:** Information on samples from different elevations in East Pamir.

Group	L	M	H
Elevation (m)	1400–2400	2400–3400	3400–4400
UV (mw/cm^2^)	4.84 ± 0.94b	6.47 ± 0.93a	7.02 ± 0.44a
Temp (°C)	25.70 ± 3.31a	23.07 ± 1.50a	17.53 ± 8.68b
Moisture (%rh)	22.87 ± 12.87a	30.32 ± 5.86a	25.67 ± 11.31a
OM (g/kg)	4.63 ± 2.52b	6.72 ± 3.66b	12.03 ± 7.51a
TN (g/kg)	0.32 ± 0.06b	0.31 ± 0.17b	0.59 ± 0.36a
AN (mg/kg)	14.67 ± 6.34c	37.44 ± 10.18a	20.67 ± 6.23b
AP (mg/kg)	5.17 ± 2.29b	10.57 ± 4.14a	8.87 ± 6.34a
AK (mg/kg)	131.67 ± 44.61a	92.00 ± 43.69a	120.67 ± 56.63a
Salt (g/kg)	33.27 ± 11.64a	3.27 ± 2.00b	7.73 ± 9.36b
pH	7.81 ± 0.08b	8.71 ± 0.28a	8.62 ± 0.18a

The above data represent the mean ± standard deviation (n = 3). Different letters (a, b) indicate significant differences between the data (Duncan test, *p* < 0.05).

**FIGURE 5:**
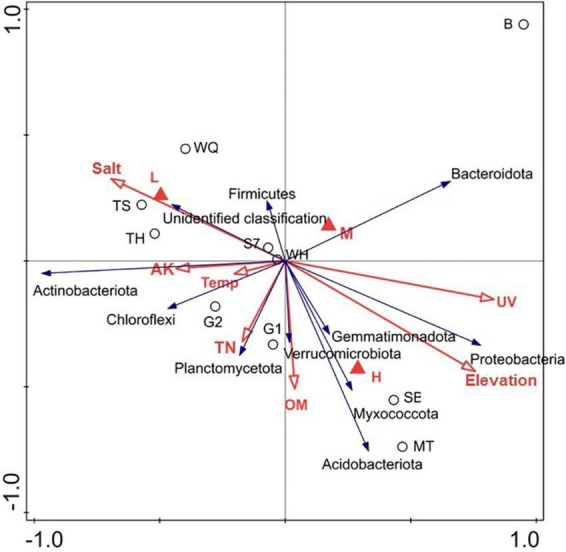
Bacterial community correlation with environmental factors

Collected environmental factor information and measured physicochemical characteristics of soil samples from the three elevations were correlated with the top 10 bacterial phyla in abundance (Figure 5). The RDA showed that 78.71% of the total variation was explained by Axis 1 and 5.45% by Axis 2. We found that UV (Contribution % = 60.9, *p* = 0.022) was the most important factor driving changes in the bacterial community. Elevation (*p* = 0.022), OM (*p* = 0.044), TN (*p* = 0.022), AK (*p* = 0.022), and Salt (*p* = 0.022) were the relevant influencing factors.

Figure 5 showed elevation was significantly and positively correlated with *Proteobacteria*, *Bacteroidetes*, *Gemmatimonadota*, *Verrucomicrobiota*, *Myxococcus*, *Acidobacteriota*, and *Planctomycetota*. UV radiation was significantly and positively correlated with *Proteobacteria*, *Bacteroidetes*, *Gemmatimonadota*, *Verrucomicrobiota*, *Myxococcus*, and *Acidobacteria*. OM was significantly and negatively correlated with *Firmicutes*, *Bacteroidetes*, and unidentified classifications. TN was negatively correlated with *Firmicutes* and *Bacteroidetes*. Temperature was negatively correlated with *Firmicutes*, *Bacteroidetes*, and *Proteobacteria*. AK was negatively correlated with *Verrucomicrobiota*, *Planctomycetota*, *Myxococcus*, and *Acidobacteria*. *Planctomycetota*, Chloroflexi, *Actinobacteria*, unidentified classification, and *Firmicutes*. Salt was negatively correlated with *Chloroflexi*, *Actinobacteria*, and a unidentified classification, whereas *Firmicutes* was positively correlated. The low elevation samples had the highest correlation with Salt, while the high elevation samples were positively correlated with factors including elevation and OM.

### 3.5 Bacterial symbiotic network and predictive analysis

**FIGURE 6:**
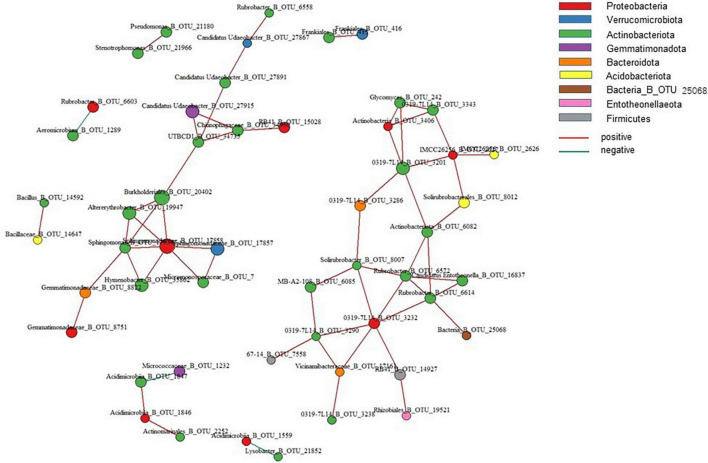
Co-occurrence relationships for the top 100 OTUs networks by abundance.

Network co-occurrence showed the ecological relationships among species in the microbial communities. Screening the top 100 OTUs for species abundance with a correlation coefficient of 0.8 and *P* = 0.05 (Figure 6), the results showed that positive correlations arose among 11 OTUs belonging to three phyla, namely, *Proteobacteria*, *Actinobacteria*, and *Gemmatimonadota*. OTUs with the highest betweenness and closeness centralities were annotated as *Sphingomonas*, *Gemmatimonadaceae*_B_OTU_8822, and *Solirubrobacter*. These OTUs were located on the lines of other nodes. These changes resulted in relevant OTU variations. Therefore, OTUs are the core microorganisms in the bacterial co-occurrence network, and changes in them caused significant changes in microbial communities.

**FIGURE 7:**
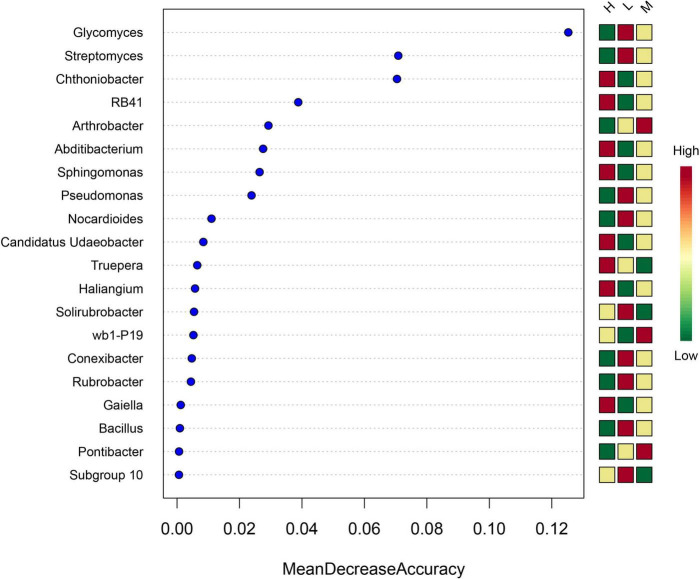
Importance of species (variable) points for the top 20 soil bacterial genera as affected by elevation.

The random forest importance of species (variable) points showed that the genera *Glycomyces*, *Streptomyces*, and *Chthoniobacter* had the highest importance of species (variable) points across soil samples (Figure 7).

### 3.6 Function pathways of soil bacteria

**FIGURE 8:**
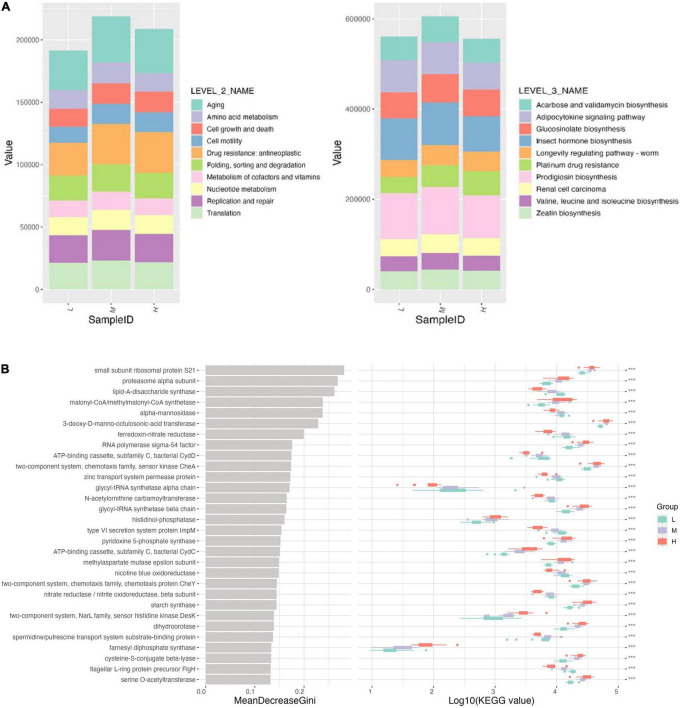
**(A)** Function profiling showing divergences in rare and abundant bacterial functions at KEGG module levels 2 and 3. **(B)** Functional pathway importance of the top 30 pathways affected by elevation.

Functional prediction of microbial communities using the PICRUSt2 database reflects microbial community function to a certain extent. A total of 10 major functional classifications were obtained on the KEGG secondary classification (Figure 8A). The top three were aging, drug resistance, antineoplastics, replication, and repair. Most of these pathways were enhanced at mid-elevations and reduced at higher elevations but were still stronger than at lower elevations. At Level 3 KEGG, the top three pathways were procyanidin biosynthesis, insect hormone biosynthesis, and adipocytokine signaling, which were inhibited at high elevations. Platinum drug resistance pathways increased with elevation. Other pathways increased at mid-altitudes, with no significant difference between low and high elevations (Figure 8A).

In Random Forest, the small subunit ribosomal protein S21, proteasome alpha subunit, and lipid-A-disaccharide synthase were the most important for maintaining the stability of the bacterial community at different elevations (Figure 8B). Meanwhile, each pathway differed significantly among the elevations.

### 3.7 Diversity of potentially radiation-resistant bacteria

**FIGURE 9:**
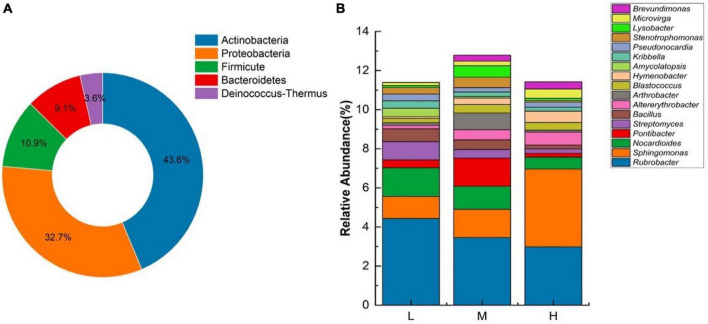
**(A)** Radiation-resistant bacterial community composition at different elevations on the Eastern Pamir Plateau based on the phyla level. **(B)** Radiation-resistant bacterial community composition at different elevations of the Eastern Pamir Plateau based on genus level.

Based on the results of previous studies, we selected 55 genera belonging to five bacterial phyla that have been reported to have radiation resistance potential (Figure 9A). Among them, *Actinobacteria* had high diversity with 24 genera, followed by *Proteobacteria* with 18 genera, *Firmicutes* with six genera, *Bacteroidetes* with five genera, *Deinococcus–Thermus* with two genera, *and Deinococcus* and *Truepera*. These two genera include bacteria that are resistant to harsh environments, such as *Deinococcus radiodurans*, which is a radiation-resistant microorganism.

The top 10 potentially radiation-tolerant microorganisms distributed at each elevation were selected and mapped to their relative abundance (Figure 9B). The top five genera in terms of abundance of potential radiation-tolerant microorganisms were *Rubrobacter*, *Sphingomonas*, *Nocardioides*, *Pontibacter*, and *Streptomyces*. Species abundance varied greatly between elevations. At low elevations, *Rubrobacter*, *Nocardioides*, and *Sphingomonas* are the dominant genera. At the middle elevations, the dominant genera were *Rubrobacter*, *Sphingomonas*, and *Pontibacter*. At high altitudes, the dominant genera were *Sphingomonas* and *Rubrobacter*. The abundance of *Rubrobacter*, *Nocardioides*, and *Streptomyces* genera showed a decreasing trend at 4.43–2.98%, 1.47–0.61%, and 0.94–0.22%, respectively. The abundance of *Sphingomonas* genera showed an increasing trend (1.13–3.98%) among different elevations. The *Pontibacter* relative abundance initially increased, and then decreased. *Deinococcus* and *Truepera* were also detected at different elevations with abundances ranging from 0.0018–0.0039% to 0.10–0.16%, respectively. Because the dominant genera in the samples from different elevations were potentially radiation-resistant, this region contains rich resources for radiation-resistant microorganisms.

**FIGURE 10:**
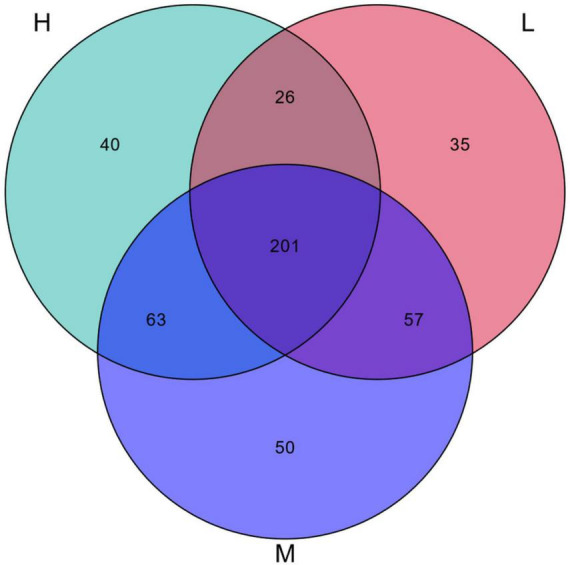
Venn diagram of OTUs of potentially radiation-resistant bacteria.

A Venn diagram showed that the distribution of OTUs differed across habitats (Figure 10). A total of 472 OTUs sequences were obtained, of which 201 were common among the three elevations, accounting for 42.58% of the total. The numbers of OTUs specific to the low, medium, and high elevations were 35 (7.42%), 50 (10.59%), and 40 (8.47%), respectively. This indicated that the diversity of radiation tolerant species was greater at the middle elevations.

**FIGURE 11:**
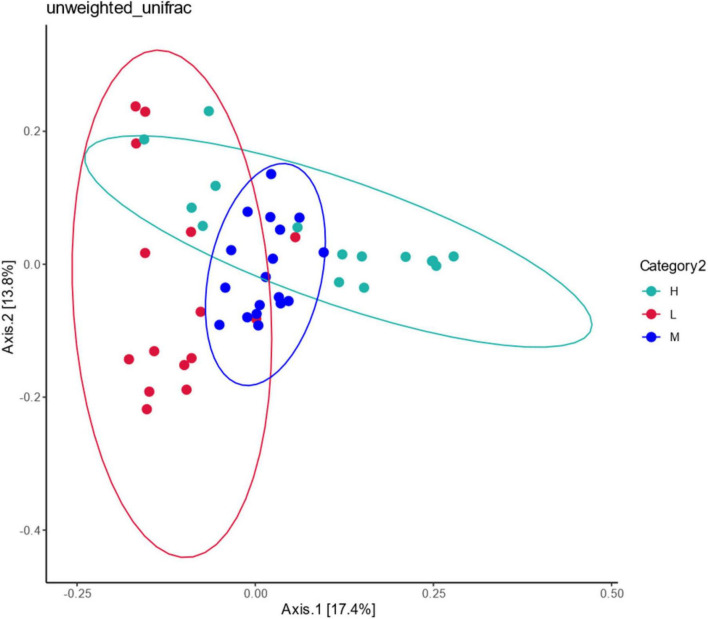
Principal coordination analysis of potentially radiation-tolerant bacteria at different elevations in East Pamir.

The correlation of community composition across sites was analyzed using Unfaire Bray–Curtis PCoA (Figure 11). Principal components 1 (PCoA1) and 2 (PCoA2) explained 17.4% and 13.8% of the radiation-tolerant bacteria in the soil samples, respectively. The samples were spatially overlapping, indicating that the radiation-resistant species were widely distributed among the three altitude zones. Only some strains were distributed at low, middle, and high elevations, and most radiation-resistant microorganisms survived more stably at different elevations, forming a stable core system.

## 4 Discussion

Microorganisms are the main drivers of biochemical cycles, and their community structure influences ecosystem stability. Plateau regions have limited soil nutrients, fragile ecosystems, and harsh environments. The Pamir Plateau is located in central Asia, with a total glacial coverage of approximately 12,260 km^2^ (Yao et al., 2012). It mainly has cold mountainous deserts. Low vegetation cover, scarce precipitation, and poor soil nutrient concentration and water content result in an extremely arid environment (Mętrak et al., 2015, 2017). To date, prior studies on microorganisms in plateau regions have focused on the Tibetan, Loess, and Mongolian Plateaus. These studies have involved variations in soil enzyme activities and microbial communities along elevation gradients (Fan et al., 2021), reflections of functional microorganisms in soil to natural factors such as nitrogen deposition and precipitation (Xu et al., 2021; Li et al., 2023), or different responses of soil microorganisms from different plant communities to changes in environmental stresses (Xu et al., 2023). Relatively few studies have been conducted on the distribution of bacterial communities in East Pamir.

Under the influence of environmental conditions, soil microorganisms adapt to the environment by regulating their community structure (Zeng et al., 2021). Zhang et al. (2021) studied bacterial diversity in desert habitats in the Tibetan Plateau region at an average altitude of 2800 m, where *Proteobacteria*, *Actinobacteria*, *Bacteroidetes*, and *Chloroflexi* were the dominant phyla. Cui et al. (2019) studied the microbial diversity of alpine ecosystems in the Tibetan Plateau at 2800–3500 m and found that the dominant phyla included *Acidobacteria* and *Proteobacteria*, whereas *Actinobacteria*, *Bacteroidetes*, and *Gemmatimonadota* were the dominant bacterial phyla. In this study, *Actinobacteria* was the dominant phylum, followed by *Proteobacteria*, *Bacteroidetes*. *Acidobacteria* and *Gemmatimonadota* were the secondary dominant phyla, which is in line with the results of Cui et al. (2019) and Zhang et al. (2021), with the same results but with differences in species abundance. *Rubrobacter*, *Sphingomonas*, *Nocardioides*, and *Solirubrobacter* were the dominant genera at the genus level. There were differences in the dominant genera among different elevations, which may be mainly because of the ecology of the sampling area, geographic location, climate change, and anthropogenic activities (Mou et al., 2020).

There are three main patterns of relationships among species diversity, community structure, and elevation: monotonically decreasing (Ding et al., 2023), humping (Nottingham et al., 2018), and U-shaped (Shen et al., 2020). Many studies have shown that Alpha diversity decreases with elevation, whereas others have reported the greatest diversity at mid-elevations, or insignificant elevation patterns (Yao et al., 2017). Ogwu et al. (2019) found that microbial abundance increased with elevation in a study on alpine microorganisms in Japan. Shen et al. (2013) found that soil bacterial diversity did not vary with elevation in the Changbai Mountain region. In contrast, the present study found differences in bacterial community diversity at different elevation, with an increasing trend with increasing elevation. Although the data described vary, mechanisms driving the different elevation patterns have not been explicitly discussed. Yao et al. (2017) revealed that the elevational patterns of dominant soil microbial taxa along Changbai mountain slope were shaped more by vegetation and soil nutrient status. This suggests that discussion about soil community structure and species diversity at different elevations also needs to incorporate the effects of environmental factors.

Elevation and soil are the main factors driving soil microbial communities (Tang et al., 2020). Bacterial communities respond to environmental factors in a complex manner, with pH usually being the main influence (Griffiths et al., 2011; Bahram et al., 2018). In addition, factors such as soil temperature, soluble salt, nitrogen, Organic matter, and precipitation also affect the composition of soil bacterial communities. Zhao et al. (2022) studied soil characteristics and microbial communities along an altitudinal gradient (1700–2300 m) in a Huashan pine forest in the Qinling Mountains and found that in addition to altitudinal factors, pH, soil temperature, OM, and AK were important factors influencing microbial communities. Wang et al. (2022) found that differences in bacterial community composition at the OTU level in soils and lake sediments from the lakeshore of Kekexili with a mean elevation of 4600 m were mainly determined by the mean annual temperature, salinity, total organic carbon, and TN content. This finding is similar to that of the present study. The environmental factors in this study were significantly distributed at different elevations and had a significant influence on microbial community composition. In study of the effects of UV on microorganisms, Natarajan et al. (2008) found the melanin content of *Aspergillus niger* spores on south slopes with strong UV radiation was three times higher than on shaded north slopes (south slopes have 2–8 times more sunlight than north slopes) in the “Evolution Canyon”, Mount Carmel, Israel. In this study, pH did not show a significant correlation to community composition. However, UV, OM, temp, TN, AK, and Salt were environmental factors affecting the bacterial community in the Pamir region and were significantly correlated with the major bacterial phyla. Low altitude samples showed the strongest correlation with Salt, whereas high elevation samples showed a positive correlation with factors such as elevation and OM.

Environmental factors at low elevations are highly variable due to human activities. Meanwhile, environmental constraints at high elevations, such as low temperatures, dryness, and high radiation intensity, lead to fragile ecosystems (Nottingham et al., 2018; Portero et al., 2019). The co-occurrence network among microorganisms reflects the function and stability of a biome (Ma et al., 2020). The co-occurrence network was used to analyze the key interactions between microorganisms and their response to the environment, examine the fundamentals of microbial communities in different soil environments and identify key hypothetical taxa in the community. In this study, 11 OTUs in the co-occurrence network belonged to three phyla, *Proteobacteria*, *Actinobacteria*, and *Gemmatimonadota*, of which *Sphingomonas*, *Gemmatimonadaceae*_B_OTU_8822, *Solirubrobacter* were in a central position as core microorganisms in the symbiotic network. Gene function prediction revealed differences in gene pathways between soil samples from neighboring elevations. Random forest results also showed that the small subunit ribosomal protein S21, proteasome α-subunit, and lipid-A-disaccharide synthase were the most important factors in maintaining the stability of the bacterial community between altitudes.

Since 1956, when DR was isolated from canned meat by Andersen et al. (1956), which deteriorated after radiation sterilization (Andersen et al., 1956), radiation-resistant microbial resources have attracted the attention of researchers. More than 1500 strains of radiation-resistant microbial strains have been identified. The isolation sources are mainly deserts, radiation-contaminated areas, hot springs, and marine sediments, which are seldom found in highland areas. In this study, we predicted and screened out species with a potential radiation tolerance function based on the currently reported genera of radiation tolerant bacteria and revealed their distribution at different elevations. The results showed that *Rubrobacter*, *Sphingomonas*, *Nocardioides*, *Pontibacter* and *Streptomyces* were the dominant genera among the potentially radiation-tolerant species in the Pamir Plateau, while *Rubrobacter*, *Sphingomonas* and *Nocardioides* were the dominant genera in the Pamir Plateau at different altitudes. This suggests this region is rich in potential radiation tolerant microbial resources. In the bacterial co-occurrence network, the genera *Rubrobacter* and Sphingomonadaceae, which are highly abundant, have been reported to be radiation tolerant. Therefore, it is assumed that the strains from this habitat also have a high potential for radiation tolerance.

## 5 Conclusion

A study of bacterial communities in soil samples from different elevations of the Pamir Plateau showed that *Actinobacteria* was the dominant phylum, whereas *Proteobacteria*, *Bacteroidetes*, *Acidobacteria*, and *Gemmatimonadota* were the subdominant phyla. The genera were dominated by *Rubrobacter*, *Sphingomonas*, *Nocardioides*, and *Solirubrobacter*. Species diversity increased slightly with elevation, whereas community specificity increased with elevation. Elevation and UV radiation were the main factors affecting the bacterial communities. Under different environmental conditions at different elevations, soil bacteria can adapt to their environment by altering KEGG pathways. The soil samples from different elevations were rich in typical radiation-tolerant resources. The main genera were *Rubrobacter*, *Sphingomonas*, *Nocardioides*, *Pontibacter*, and *Streptomyces*. The composition of radiation-tolerant bacterial communities varied significantly among different elevations. The results of this study have shown the composition and distribution of bacterial communities at different elevations on the Pamir Plateau. It is of considerable importance to examine the potential genera of radiation-resistant microorganisms. This will be important for the succession of bacterial microorganisms on the plateau and for the study of radiation-resistant microbial germplasm resources and their biofunctional applications.

## Data availability statement

The datasets presented in this study have been deposited in the Sequence Read Archive of the National Center for Biotechnology Information (SRA, NCBI, www.ncbi.nlm.nih.gov/sra) under the BioProject IDs PRJNA1032247. The ASV tables, fasta sequences, and taxonomy data for bacteria were uploaded to Figshare repository, resulting in https://doi.org/10.6084/m9.figshare.25435474.

## Author contributions

JZ: Conceptualization, Funding acquisition, Investigation, Writing–original draft, Writing–review and editing. H-NW: Formal analysis, Writing–original draft. Q-YT: Data curation, Investigation, Writing–review and editing. M-YG: Investigation, Supervision, Writing–review and editing. Z-DZ: Conceptualization, Investigation, Writing–review and editing.
